# Utilizing the Wikidata System to Improve the Quality of Medical Content in Wikipedia in Diverse Languages: A Pilot Study

**DOI:** 10.2196/jmir.4163

**Published:** 2015-05-05

**Authors:** Alexander Pfundner, Tobias Schönberg, John Horn, Richard D Boyce, Matthias Samwald

**Affiliations:** ^1^Section for Medical Expert and Knowledge-Based SystemsCenter for Medical Statistics, Informatics, and Intelligent SystemsMedical University of ViennaViennaAustria; ^2^UnaffiliatedInnsbruckAustria; ^3^School of PharmacyUniversity of WashingtonSeattle, WAUnited States; ^4^Department of Biomedical InformaticsUniversity of PittsburghPittsburgh, PAUnited States

**Keywords:** Internet, Wikipedia, drug information services, semantic networks, medical informatics, drug interactions

## Abstract

**Background:**

Wikipedia is an important source of medical information for both patients and medical professionals. Given its wide reach, improving the quality, completeness, and accessibility of medical information on Wikipedia could have a positive impact on global health.

**Objective:**

We created a prototypical implementation of an automated system for keeping drug-drug interaction (DDI) information in Wikipedia up to date with current evidence about clinically significant drug interactions. Our work is based on Wikidata, a novel, graph-based database backend of Wikipedia currently in development.

**Methods:**

We set up an automated process for integrating data from the Office of the National Coordinator for Health Information Technology (ONC) high priority DDI list into Wikidata. We set up exemplary implementations demonstrating how the DDI data we introduced into Wikidata could be displayed in Wikipedia articles in diverse languages. Finally, we conducted a pilot analysis to explore if adding the ONC high priority data would substantially enhance the information currently available on Wikipedia.

**Results:**

We derived 1150 unique interactions from the ONC high priority list. Integration of the potential DDI data from Wikidata into Wikipedia articles proved to be straightforward and yielded useful results. We found that even though the majority of current English Wikipedia articles about pharmaceuticals contained sections detailing contraindications, only a small fraction of articles explicitly mentioned interaction partners from the ONC high priority list. For 91.30% (1050/1150) of the interaction pairs we tested, none of the 2 articles corresponding to the interacting substances explicitly mentioned the interaction partner. For 7.21% (83/1150) of the pairs, only 1 of the 2 associated Wikipedia articles mentioned the interaction partner; for only 1.48% (17/1150) of the pairs, both articles contained explicit mentions of the interaction partner.

**Conclusions:**

Our prototype demonstrated that automated updating of medical content in Wikipedia through Wikidata is a viable option, albeit further refinements and community-wide consensus building are required before integration into public Wikipedia is possible. A long-term endeavor to improve the medical information in Wikipedia through structured data representation and automated workflows might lead to a significant improvement of the quality of medical information in one of the world’s most popular Web resources.

##  Introduction

Wikipedia is an important source of medical information for both patients and medical professionals [1,2]. Medical Wikipedia content is made up of more than 150,000 articles in 255 languages [3]. Given its wide reach, improving the quality, completeness, and accessibility of medical information on Wikipedia could have a positive impact on global health. For example, adverse events caused by drug-drug interactions (DDIs) are a significant cause of morbidity [4], but adverse event data on Wikipedia is currently incomplete in ways that might cause harm to patients [5-7].

To address these issues, we created a prototypical implementation of an automated system for keeping drug information in Wikipedia up to date with current evidence about clinically significant drug interactions. Our work is based on Wikidata [8,9], a novel database backend of Wikipedia currently in development. Wikidata is a large, community-edited, multilingual graph database aiming to capture facts in a centralized store that can be dynamically queried and displayed by different language versions of Wikipedia. As such, Wikidata could emerge as a community-backed and highly visible structured knowledge base of medical and biological information, bringing concepts and methodologies such as controlled taxonomies, Semantic Web / semantic technologies and ontologies into mainstream use.

**Figure 1:**
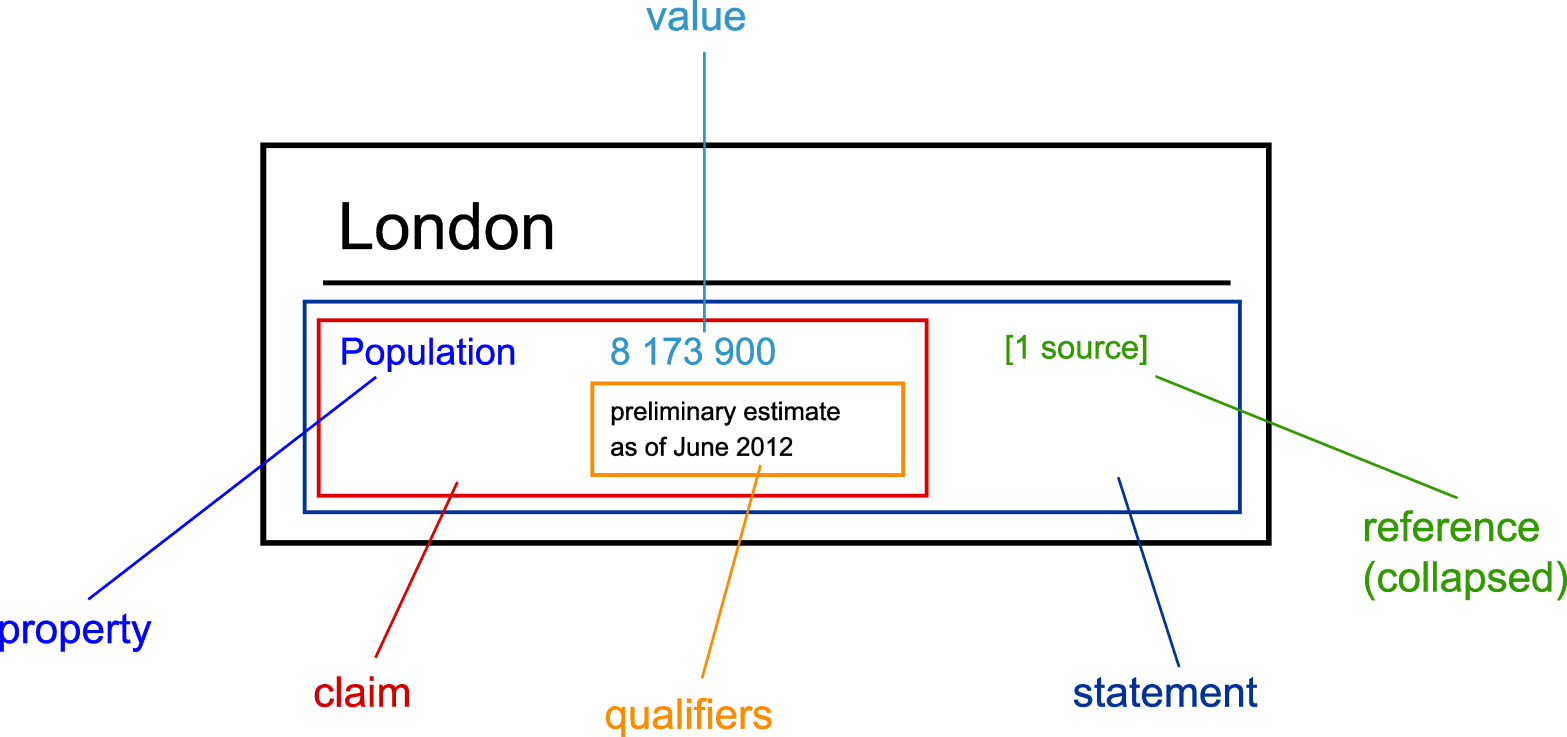
The structure of a statement in Wikidata. Source: [10].

The data model of Wikidata is exemplified in Figure 1. The model differs from that of common databases in that information is captured through property-value pairs and each entry is individually sourced. A property resembles a field for a specific piece of information (eg, the population of a city). To complete the pair, a value is added for this property (eg, a population count of 8,173,900). Together this pair is called a *claim* and it is the smallest amount of information that can be added to Wikidata. A claim can also contain qualifiers that put the value into context (eg, the year of the population estimate is given). Each claim can have a multitude of qualifiers. The practice of using claims (instead of “facts”) reflects the notion that Wikidata anticipates and embraces situations in which conflicting claims are made when different sources might disagree. An advantage of this data model is that only claims with sources are considered as complete statements and that disagreement in the sources can be modeled by adding multiple statements.

At the time of this writing (November 2014), Wikidata contained 50 million statements about more than 12 million data items and had more than 13,000 active participants. However, the large-scale participative character of Wikidata and its integration with Wikipedia also lead to unprecedented challenges. In this paper, we describe a pilot project utilizing the Wikidata system to improve information on DDIs in Wikipedia. The aim of this pilot is to provide insights into strategies for improving drug safety data in Wikipedia and provide guidance on utilizing the Wikidata system for capturing, sharing, and accessing biomedical knowledge in general.

## Methods

### Drug-Drug Interaction Data

We reviewed openly available DDI knowledge bases, including Drugbank [10], the National Drug File-Reference Terminology (NDF-RT) [11], a short list of clinically important and common DDIs maintained by the CredibleMeds nonprofit organization [12], the Office of the National Coordinator for Health Information Technology (ONC) “high priority” list of potential DDIs suggested as high priority to alert clinicians in any care environment [13], and the ONC “noninterruptive” list of potential DDIs not requiring interruptive alerting in any care environment [14]. We decided to include only clinically relevant potential DDIs that had high priority and were curated by teams of experts in order to avoid overloading the knowledge base with large numbers of interactions that were not critical or not backed by sufficient evidence [14]. We selected the ONC high priority list for these reasons, reserving the possibility of further extension of the knowledge base at a later point in time.

### Adding Data to Wikidata and Wikipedia

The process for adding potential DDI data to Wikidata consisted of several steps and community interactions. First, we registered for accounts on the Wikidata system. In general, every person with or without an account can directly edit data pages via the Wikidata website. Complex contribution tasks are usually organized through WikiProjects (eg, WikiProject Medicine), which are the first points of contact for people who share a specific goal in Wikidata development. If users want to be part of a WikiProject, they simply have to add themselves to the project participant list. We joined the Wikidata Medicine project [15].

We researched if appropriate Wikidata properties currently existed or if it would be necessary to create a new Wikidata property. We found no existing property for capturing potential DDIs, so we drafted a proposal for a new property and submitted it for community discussion and voting (discussion archived at [16]). Three discussants participated in this discussion. After the name, description, and semantics were refined by us based on community feedback and the utility and novelty of the property were ascertained, the proposed property was accepted and added to the Wikidata system [17]. Major discussion points were the inclusion of food-drug interactions, whether the property should be symmetric or should be nonsymmetric to discern precipitant and objects drugs, and whether existing properties for physical interactions between objects could be reused. The entire process took 1 week. Minor backwards-compatible refinements to the property definition were conducted at later stages of the process based on requirements that became apparent during data loading. After a property is created, any user can translate it. Additionally, property constraint can be set that help to find mistakes in the data when such constraints are violated.

We created a Wikidata “bot” (a program that autonomously adds or edits content) for automatically adding and updating interactions from the larger ONC high priority list. The implementation and application of the bot was discussed with the Wikidata community until consensus on its utility and the scope of the imported data was reached (discussion archived at [18]). Eight discussants participated in this discussion.

To operate the bot, we registered a separate bot account and created a request for permission (as stated in the Wikipedia bot policy [19]). The request’s text included a description of our team and the bots main functionality and goals. The resulting discussion included 8 other Wikipedians (mainly members of the WikiProject Medicine) and spanned more than a month before the bot was approved by a Wikidata “bureaucrat” (the official title of members responsible for such decisions in the Wikipedia/Wikidata community). The bot was created by using the Pywikibot framework, which is a collection of Python scripts for maintaining different MediaWiki sites [20]. We derived the bot from a basic template script [21], which is part of the Pywikibot software package and used specific functionality geared toward Wikidata editing. The code of the bot we created is available on GitHub [22].

We created modified templates of drug infoboxes for the English and German Wikipedia that utilize potential DDI data from the Wikidata knowledge base; Infoboxes are templates that typically accompany a Wikipedia article and are usually displayed at the top-right of a page. They typically contain the most important facts about a Wikipedia entry. We copied selected Wikipedia articles into an online development environment and integrated the modified drug infobox templates so that potential DDI data from Wikidata were automatically shown in the resulting Wikipedia articles. This workflow can potentially be implemented for any of the more than 288 languages of Wikipedia as long as the property name and the statement’s values are translated. These articles were used as a proof of concept and to predict how information from Wikidata would be displayed in Wikipedia with current technologies.

For this pilot study, we implemented these changes in a segregated Wikipedia development environment instead of the main system because changes to widely used templates (eg, the drug infobox) usually require extensive discussion. Given that the Wikidata application program interface (API) is still not fully stable, large-scale adoption of Wikidata-based infoboxes are not yet approved by the community. The Wikipedia development environment is not technically different from normal Wikipedia, but it is less visible (eg, Web search engines do not usually point to pages in the development environment).

### Analysis

We conducted a pilot analysis to explore if adding the ONC high priority data would significantly enhance the information currently available on Wikipedia in terms of coverage of significant potential drug interactions. We developed a simple script that analyzed English Wikipedia articles of substances in the ONC high priority list and checked if interacting substances were mentioned in the current Wikipedia article through string matching.

Finally, an expert clinical pharmacist (JH) conducted a detailed evaluation of the current Wikipedia DDI information available for 2 examples of drugs on the ONC list (ramelteon and warfarin). A standard reference for clinically important DDIs coauthored by the expert was used as basis for the evaluation [23].

## Results

**Figure 2:**
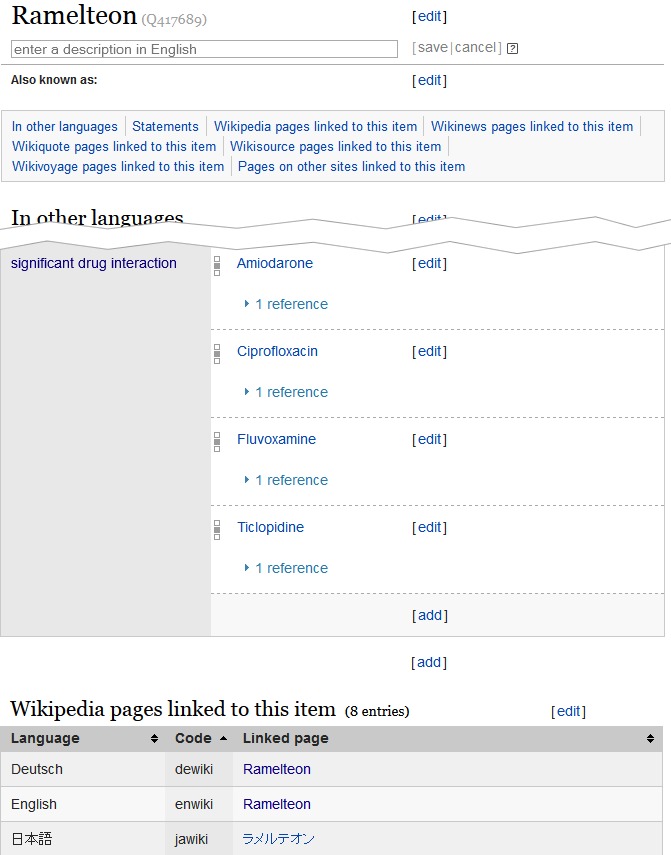
Screenshot of drug interaction data for the pharmaceutical ramelteon in the Wikidata system. A large number of other properties not pertaining to drug-drug interaction data have been removed from the screenshot.

**Figure 3:**
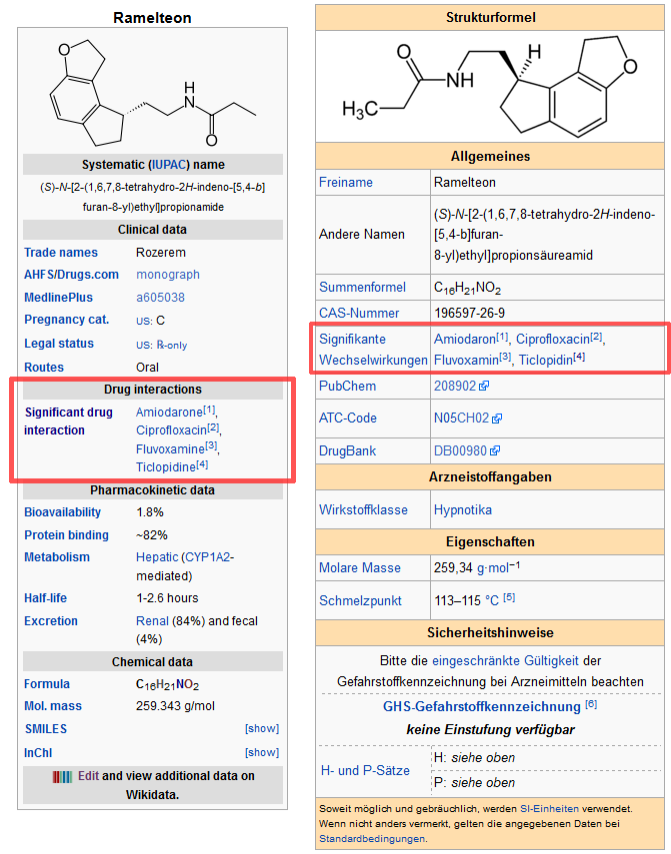
Screenshots of the English and German infoboxes of the pharmaceutical ramelteon. Drug interaction data automatically queried from the Wikidata knowledge base is highlighted.

We derived 1150 unique interactions from the ONC high priority list. The coverage of active ingredients in Wikidata was exhaustive and we did not need to create new entities. A screenshot of an example of the data represented in Wikidata is shown in Figure 2 and screenshots of the same data as represented in an infobox in the German and English Wikipedia are shown in Figure 3.

**Figure 4:**
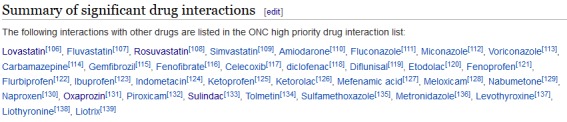
Screenshot of a long list of drug interaction data from Wikidata embedded into the main text of a Wikipedia article. The example shows drugs interacting with warfarin.

Integration of the potential DDI data from Wikidata into the Wikipedia infobox proved to be straightforward and yielded useful results, but also highlighted potential shortcomings of the current system. We recognized difficulties with making long lists of interacting drugs accessible through the infoboxes because some drugs had interactions with up to 30 other drugs. This problem could be addressed by hiding long lists of interacting drugs behind expandable user interface elements, which is already done for other use cases (eg, gene ontology term annotations on protein articles), or by removing other information that might be of lower interest. Still, the existence of these long lists of interactions for some drugs might lead to unsatisfactory usability or reduced findability of important information. An alternative could be the integration of the Wikidata information into the main text of the Wikipedia article as exemplified in Figure 4.

Another shortcoming of the current integration of Wikidata into Wikipedia that was uncovered by our prototype is the way in which literature citations/evidence is rendered. Although backing evidence from Wikidata can be displayed through endnote references in Wikipedia, the Wikipedia system currently does not recognize that multiple citations may point to a single reference, leading to the creation of redundant reference list entries. Furthermore, the current system is only able to display the titles of literature references, but does not provide a formatted endnote with all bibliographic details (eg, authors, journal, and publication year). These current limitations reduce the ability of Wikipedia readers to efficiently check the evidence behind data from the Wikidata knowledge base. However, given the novelty of Wikidata and the ongoing development of the database interfaces, this limitation will soon disappear.

### Statistics on Drug-Drug Interaction Information in Wikipedia

We found that even though the majority of current English Wikipedia articles about pharmaceuticals contained sections detailing contraindications, only a small fraction of articles explicitly mentioned interaction partners from the ONC high priority list in the article text (Table 1). For 91.30% (1050/1150) of the 1150 interaction pairs we tested, none of the 2 articles corresponding to the interacting substances explicitly mentioned the interaction partner. For 7.21% (83/1150) of the pairs, only 1 of the 2 associated Wikipedia articles mentioned the interaction partner, and for only 1.48% (17/1150) of the pairs both articles contained explicit mentions of the interaction partner.

**Table 1:** Statistics on coverage of drug-drug interaction (DDI) data in existing English Wikipedia articles compared to the ONC high priority list for 1150 DDI pairs tested.

Coverage	n (%)
Both articles of interacting pair contain explicit mention of interaction partner (symmetric occurrence). Example: amiodarone-simvastatin	17 (1.48)
Only 1 article of interacting pair contains explicit mention of interaction partner (asymmetric occurrence) Example: fluoxetine-phenelzine	83 (7.21)
None of the 2 articles of interacting pair contain explicit mention of interaction partner	1050 (91.30)

A review of randomly selected articles showed that many contained implicit information about drug interactions by providing information about interacting drug classes or interactions with enzymes. However, in many cases, readers might not have the background knowledge necessary to infer actionable information from these statements (eg, the warning that a drug significantly inhibits the cytochrome P450 3A4 [CYP3A4] enzyme requires the knowledge that a potentially coadministered drug is metabolized by CYP3A4 to be of practical utility). We also observed that many articles that did not contain explicit mentions of interacting drugs from the ONC high priority list did mention interactions with other drugs that were not on the list, suggesting a significant overall discordance between drug interaction information on Wikipedia and the ONC high priority list.

It is also important to keep in mind that the ONC list is not intended to be inclusive of all clinically relevant DDIs because it was developed from a short list of interacting drug pairs and then expanded to include related drugs that also interact. This makes the lack of DDIs in Wikipedia even more disturbing because many important DDIs are simply missing.

### Detailed Analysis of Exemplary Wikipedia Drug Articles

The following detailed analyses of DDI information in Wikipedia articles for the drugs ramelteon and warfarin exemplify some of the limitations of the current Wikipedia article coverage of DDIs from the perspective of a professional pharmacologist.

As noted in Figure 3, ramelteon is primarily metabolized by CYP1A2. CYP3A4 and CYP2C9 also contribute to its metabolism. Thus, any drug that alters the activity of CYP1A2, CYP3A4, or CYP2C9 is likely to alter the elimination of ramelteon. Ramelteon also has a very low bioavailability (amount of oral drug reaching systemic circulation) of 1.8%. This means that if a 10 mg dose of ramelteon is taken orally, less than 0.2 mg will reach the systemic circulation and produce a pharmacologic response. If another drug inhibits the metabolism of ramelteon, the amount of ramelteon reaching the systemic circulation will be increased, potentially to a very large extent.

Currently, Wikipedia notes several drugs that do not interact with ramelteon; however, it is not clear if the drugs have no effect on ramelteon or that ramelteon has no effect on the listed drugs. Actually, both outcomes are true. None of the drugs listed as noninteracting with ramelteon would be expected to interact because they do not affect any of the metabolic pathways that metabolize ramelteon. The Wikipedia entry further states:

A drug interaction study showed that there were no clinically meaningful effects or an increase in adverse events when ramelteon and the SSRI Prozac (fluoxetine) were coadministered. Ramelteon and fluvoxamine should not be coadministered. Ramelteon should be administered with caution in patients taking other CYP1A2 inhibitors, strong CYP3A4 inhibitors such as ketoconazole, and strong CYP2C9 inhibitors such as fluconazole. Efficacy may be reduced when ramelteon is used in combination with potent CYP enzyme inducers such as rifampin, since ramelteon concentrations may be decreased.

Although no references are provided for the preceding statements, they appear to be taken from the ramelteon product label. We agree that ramelteon should be avoided in patients taking fluvoxamine because the label notes a 190-fold increase in ramelteon levels resulting from the interaction. Fluvoxamine inhibits CYP1A2 as well as CYP2C9 and CYP3A4. As noted previously, CYP1A2 is the primary enzyme that metabolizes ramelteon. Other drugs that inhibit CYP1A2 include atazanavir, ciprofloxacin, amiodarone, enoxacin, mexiletine, tacrine, thiabendazole, cimetidine, ticlopidine, zileuton, and vemurafenib, yet none of these are mentioned in the Wikipedia article. Likewise, there are many drugs that inhibit both CYP3A4 and CYP2C9 other that the 2 drugs listed in the article. For example, atazanavir, ciprofloxacin, and cimetidine inhibit 2 of the metabolic pathways of ramelteon and would potentially cause large increases in ramelteon plasma concentrations.

Warfarin is primarily metabolized by CYP2C9 with CYP1A2 and CYP3A4 also contributing to its elimination. Although too lengthy to reproduce fully here, Wikipedia notes drugs that “can displace warfarin from serum albumin and cause an increase in the international normalized ratio (INR)” can interact with warfarin. This is theoretically true; however, because the clearance of warfarin from the blood is limited to drug that is not attached to albumin, displaced warfarin is metabolized and the amount of active warfarin at steady state does not change so the INR is also unchanged.

Antibiotics such as metronidazole and macrolides are noted to “greatly increase the effect of warfarin” by reducing its metabolism. Metronidazole will reduce the metabolism of warfarin, as will several other antibiotics and antifungal agents that are not mentioned in the article and are likely to be more commonly used and produce larger changes in warfarin concentrations than the drugs cited. Macrolides do not appear to alter warfarin metabolism. They have been associated with enhanced warfarin effect, but this is likely due to other causes including the infection itself or altered diet. Several cases of altered warfarin response associated with thyroid activity are also cited in the article. It has been demonstrated that the administration of thyroid supplementation does not alter patients’ response to warfarin. The Wikipedia article notes that excessive use of alcohol can increase the response to warfarin. This is true for binge drinking; however, chronic alcohol consumption is likely to increase the metabolism of warfarin leading to reduced response.

Several paragraphs in the article recount selected reports of herb-warfarin interactions. Most of the reports cited do not meet even minimal standards for evidence of an interaction and none of the trials showing no or minimal effects of the herbals on warfarin are included. This is an example of selection bias in DDI reporting.

As with the ramelteon article, the warfarin article omits many important DDIs. No mention is made of potent inhibitors of CYP2C9, such as amiodarone, sulfamethoxazole, fluconazole, voriconazole, or fluoxetine. No mention is made of drugs that can increase the risk of bleeding in patients taking warfarin, such as the nonsteroidal antiinflammatory drugs or acetaminophen.

## Discussion

### Principal Results

We implemented a process for enriching medical data in the Wikidata knowledge base and demonstrated that automated updating of medical content in Wikipedia through Wikidata is a viable option, albeit further refinements and community-wide consensus building are required before integration into public Wikipedia is possible. Adding data to Wikidata and Wikipedia is a lengthy process that requires lots of community interactions and familiarity with customs and requirements of the respective communities. We expect that actual integration into the self-governed Wikipedias in each language will require further refinement and substantial dialog with the different WikiProject Medicine communities.

Better data quality in Wikidata can reduce the maintenance work required in Wikipedia, giving editors more time to focus on the quality of articles. If an article exists in all 288 languages of Wikipedia, keeping it up to date or adding a piece of data with Wikidata amounts to a single edit compared to 288 edits without Wikidata. This helps to improve the completeness and currentness of medical content on Wikipedia, a resource that has become central to health information seeking among patients and health professionals on a global scale.

We had the experience that the Wikidata community was very open toward novel participants and provided constructive feedback and assistance with the integration of novel data into the complex Wikidata knowledge base. We decided to invite one Wikipedia member who provided significant support to become a coauthor of this manuscript (TS). Based on our experiences, we strongly recommend the inclusion of long-term Wikidata and Wikipedia community members in scientific or medical projects such as this one.

Wikidata is a recent addition to the Wikipedia ecosystem and its strengths and weaknesses in routine widespread use for serving complex data to Wikipedia or as a general-purpose knowledge base have yet to be determined. Although centralized data management in Wikidata can improve efficiency of data management and quality in Wikipedia, its integration into Wikipedia might also be a source of problems. Of special concern is the fact that not all data in Wikidata are necessarily displayed in Wikipedia and that the “many eyeballs” principle that helps to correct errors in Wikipedia might not apply to some of the content in Wikidata. The inclusion of data without long-term plans of maintenance or inclusion in visible Wikipedia articles might lead to a problematic accumulation of outdated data.

The review of the DDI entries for warfarin and ramelteon reveals the severe limitations of the current system to provide clinically important and useful DDI information. This problem might be partially mitigated by the strong community interaction and feedback that helps to decide which data to include or not include into Wikidata to keep the knowledge base manageable. Furthermore, the usage of automated bots to import and map data from primary sources into Wikidata might play an important role. The bots can also make routine checks to determine if the different Wikipedias use the same data and if the data are a subset of the data in Wikidata. Wikidata is also developing tools to check for inconsistencies in the data. Furthermore, the community is not only tending to the Wikipedia-wide data but is also encouraged to report mistakes to the source database, thereby improving databases that are willing to share their data.

When we compared explicit mentions of potential drug interactions in Wikipedia with interactions from the ONC high priority list, we found a large amount of missing information in English Wikipedia. This might be even more pronounced in other language versions of Wikipedia that have, in general, fewer editors and worse coverage than English Wikipedia. This finding resonates with prior research that found substantial differences in drug interaction pairs captured in DDI knowledge bases [24,25]. The inclusion and exclusion criteria of a DDI knowledge base are vital to its practical utility. Although failing to include highly significant DDI can have obvious negative consequences, the permissive inclusion of large numbers of DDI backed by insufficient evidence or of low clinical significance can have a negative impact as well because it can lead to cognitive overload and the inobservance of truly significant interactions [13], frustrating clinicians [26] and leading to inappropriate responses [27]. The inclusion of clinically important DDIs based on critical evaluation of primary DDI evidence or established expert-curated DDI resources should be a goal of the Wikipedia/Wikidata community.

### Limitations

The publication of medical data on a public website that includes readers from the general public requires special attention to ensure proper understanding of the data and its implications. In this regard, further work is required to reduce the risk of improper utilization of the data (eg, making it clear that nonoccurrence in the list does not imply that a certain drug combination does not carry risks of adverse events). Furthermore, when the current implementation notes a potential interaction, it does not provide further information on the mechanism of the interaction or potential actions to mitigate patient risk.

A major limitation of our current implementation is the disjoint presentation of potential DDI data from Wikidata and potential DDI information in the main text of the Wikipedia article. The potential redundancies and differences between the 2 information sources might further add to this confusion and automatic checks comparing the Wikipedias to Wikidata are not in place yet for drug interactions. In order to have all potential DDI information in one place, integrating potential DDI data as a table into main text under a “drug interactions” heading might be preferable to the inclusion in the infobox on the upper right hand of the page. Furthermore, editing policies need to be set up to clarify the relation between structured data from Wikidata and unstructured text in Wikipedia. Routine checks by bots, possibly after every edit to a drug page, could potentially determine if the interactions listed are a subset of the data in Wikidata. We will investigate the automated inclusion of structured data into the main article once the necessary features in both Wikipedia and Wikidata have reached sufficient maturity, which was not yet the case at the time of this writing.

A limitation of our evaluation methodology was that the string matching approach used for identifying DDI mentions might have missed mentions that used drug class references rather than individual substances.

### Comparison With Prior Work

The Wikidata WikiProject Medicine that we are participating in is also involved in other endeavors, such as managing sitelinks for medical topics or connecting medical topics with their corresponding identifiers in medical databases. Furthermore, a WikiProject for molecular biology content on Wikidata was recently established [28].

Wikidata might also become an interesting platform for large-scale, graph-based knowledge integration tasks in the biomedical field that have been realized with Semantic Web technologies in recent years, such as Linked Open Drug Data [29] or Bio2RDF [30]. Further research and pilot projects are needed to fathom the potential of Wikidata to become a centralized repository of large-scale medical and life science data.

### Conclusions

We believe that this work provides a foundation for a long-term endeavor to improve the medical information in Wikipedia through structured data representation and automated workflows. It will strengthen the collaboration with the medical Wikipedia community to bring high-quality information on drug safety into production use as part of Wikipedia in diverse languages. We will also seek to align our work on drug safety information in Wikidata with projects in related domains, such as biomedical research and the life sciences [31,32].

Finally, we are currently preparing a collaboration with international experts in clinical pharmacology and drug safety to establish and maintain a machine-readable, open-source representation of important DDIs based on the ONC high priority list as well as other sources. The establishment of such a resource could not only benefit the quality of drug safety data in Wikipedia, but also improve the quality of clinical decision support interventions or even drug product labels [33].

To have a sustained impact, it is vital that the Wikipedia community carries this work further in both the structured world of Wikidata and the textual world of Wikipedia. We invite interested readers to join this effort.

## Abbreviations

API: application program interface
DDI: drug-drug interactions
INR: international normalized ratio
ONC: Office of the National Coordinator for Health Information Technology
